# Poly[[di­aqua­[μ-1,4-bis(pyridin-4-ylmeth­yl)piperazine-κ^2^ *N*:*N*′]{μ-2,2′-[(1,4-phenyl­ene)bis(­oxy)]di­acetato-κ^2^ *O*:*O*′}cobalt(II)] penta­hydrate]

**DOI:** 10.1107/S1600536814008745

**Published:** 2014-05-03

**Authors:** Alexander D. Sample, Robert L. LaDuca

**Affiliations:** a Lyman Briggs College, Department of Chemistry, Michigan State University, East Lansing, MI 48825, USA

## Abstract

In the title compound, {[Co(C_10_H_8_O_6_)(C_16_H_20_N_4_)(H_2_O)_2_]·5H_2_O}_*n*_, octa­hedrally coordinated Co^II^ ions on crystallographic inversion centres are bound by *trans* O atoms belonging to two hydro­quinone-*O,O′*-di­acetate (hqda) anions {systematic name: 2,2′-[(1,4-phenyl­ene)bis­(­oxy)]di­acetate}, two *trans*-pyridine N-donor atoms from two bis­(pyridin-4-ylmeth­yl)piperazine (4-bpmp) ligands, and two *trans* aqua ligands. The exobidentate hqda and 4-bpmp ligands form [Co(hqda)(4-bpmp)(H_2_O)_2_]_*n*_ coordination polymer layers parallel to (110) that are anchored into the full crystal structure by O—H⋯O hydrogen bonding between aqua ligands and ligated hqda O atoms. Disordered water mol­ecules of crystallization occupy incipient channels along [100]. However, these could not modeled reliably and so they were treated with SQUEEZE in *PLATON* [Spek (2009 ▶). *Acta Cryst*. D**65**, 148–155]; the crystal data take the presence of these mol­ecules into account. The crystal under investigation was twinned by non-merohedry, the twin fraction of the components being 53.3% and 46.7%. Only data from the major twin component were used in the refinement.

## Related literature   

For the preparation of bis­(4-pyridymeth­yl)piperazine, see: Niu *et al.* (2001 ▶). For the preparation of divalent metal terephthalate coordination polymers containing 4-bpmp, see: Farnum *et al.* (2013 ▶).
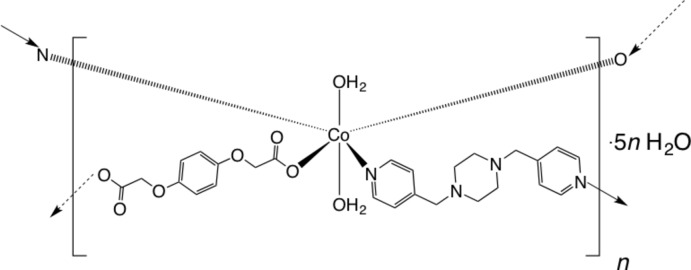


## Experimental   

### 

#### Crystal data  

[Co(C_10_H_8_O_6_)(C_16_H_20_N_4_)(H_2_O)_2_]·5H_2_O
*M* *_r_* = 677.57
Triclinic, 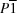

*a* = 5.7727 (8) Å
*b* = 10.3421 (15) Å
*c* = 13.1675 (19) Å
α = 87.175 (2)°
β = 78.856 (2)°
γ = 81.474 (2)°
*V* = 762.61 (19) Å^3^
*Z* = 1
Mo *K*α radiation
μ = 0.63 mm^−1^
*T* = 173 K
0.19 × 0.17 × 0.05 mm

#### Data collection  

Bruker APEXII CCD diffractometer
Absorption correction: multi-scan (*TWINABS*; Sheldrick, 2003 ▶) *T*_min_ = 0.676, *T*_max_ = 0.745
13385 measured reflections
2786 independent reflections
2032 reflections with *I* > 2σ(*I*)
*R*_int_ = 0.053

#### Refinement  

*R*[*F* ^2^ > 2σ(*F* ^2^)] = 0.093
*wR*(*F* ^2^) = 0.228
*S* = 1.10
2786 reflections
179 parameters
H-atom parameters constrained
Δρ_max_ = 0.71 e Å^−3^
Δρ_min_ = −0.89 e Å^−3^

### 

Data collection: *APEX2* (Bruker, 2012 ▶); cell refinement: *SAINT* (Bruker, 2012 ▶); data reduction: *SAINT* (Bruker, 2012 ▶); program(s) used to solve structure: *SHELXS97* (Sheldrick, 2008 ▶); program(s) used to refine structure: *SHELXL97* (Sheldrick, 2008 ▶); molecular graphics: *OLEX2* (Dolomanov *et al.*, 2009 ▶); software used to prepare material for publication: *OLEX2*.

## Supplementary Material

Crystal structure: contains datablock(s) I, pub. DOI: 10.1107/S1600536814008745/tk5308sup1.cif

Structure factors: contains datablock(s) I. DOI: 10.1107/S1600536814008745/tk5308Isup2.hkl

CCDC reference: 997809

Additional supporting information:  crystallographic information; 3D view; checkCIF report

## Figures and Tables

**Table 1:** Hydrogen-bond geometry (Å, °)

*D*—H⋯*A*	*D*—H	H⋯*A*	*D*⋯*A*	*D*—H⋯*A*
O4—H4*A*⋯O1^i^	0.91	2.28	2.945 (7)	130
O4—H4*B*⋯O2	0.91	1.85	2.636 (7)	143

Symmetry code: (i) 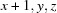.

**Fig. 1.**
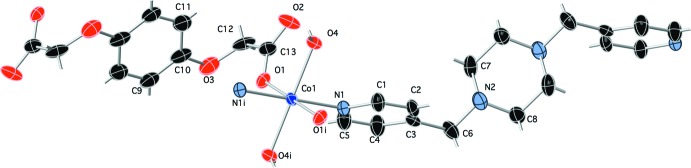
The octahedral coordination environment of the title compound, showing 50% probability ellipsoids and atom numbering scheme. Hydrogen atom positions are shown as gray sticks. Color codes: dark blue Co, red O, light blue N, black C. Symmetry code: (i) -x + 1, -y, -z.

**Fig. 2.**
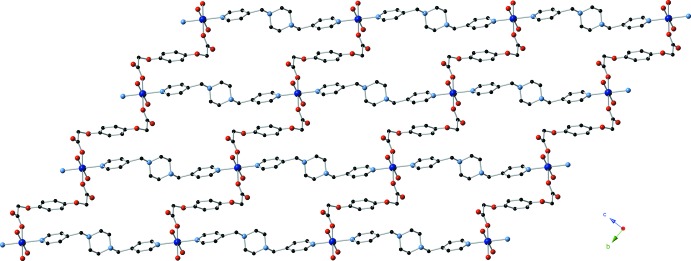
A single [Co(hqda)(4-bpmp)(H2O)2]n coordination polymer layer.

**Fig. 3.**
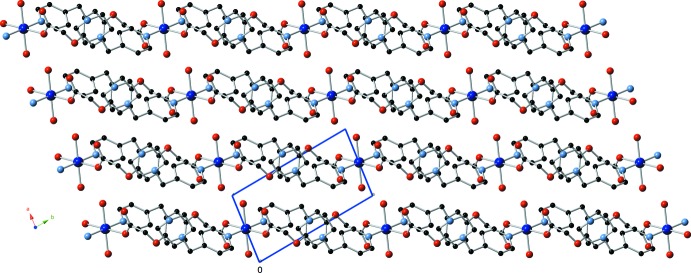
Stacking of coordination polymer layers within the title compound.

## supplementary crystallographic information

### 1. Chemical context

Some divalent metal terephthalate coordination polymers with bis­(pyridin-4-yl­methyl)­piperazine (4-bpmp) coligands show intriguing entangled topologies. (Farnum *et al.*, 2013). We hoped to expand the scope of these materials by using a *para* aromatic di­carboxyl­ate with longer pendant arms, such as hydro­quinone-*O,O'*-di­acetic acid (H_2_hqda). The title compound was obtained as pink crystals through the hydro­thermal reaction of cobalt nitrate, H_2_hqda, and 4-bpmp.

### 2. Structural commentary

The asymmetric unit of the title compound contains a divalent cobalt atom on a crystallographic inversion centre, an aqua ligand, half of a hqda ligand whose centroid rests on another crystallographic inversion centre, and one half of a 4-bpmp ligand whose centroid rests on a third crystallographic inversion centre.

The cobalt atom is o­cta­hedrally coordinated (Fig. 1), with the equatorial plane containing *trans* pyridyl N atom donors from two 4-bpmp ligands and *trans* O atom donors from monodentate carboxyl­ate groups belonging to two hqda ligands. The aqua ligands are located in the axial positions.

The Co atoms are connected by exobidentate, bis­(monodentate) hqda ligands to form [Co(hqda)(H_2_O)_2_]_n_ coordination polymer chains that are oriented parallel to [0 1 0]. Each individual chain is linked to two others by tethering 4-bpmp ligands, to construct [Co(hqda)(4-bpmp)(H_2_O)_2_]_n_ coordination polymer layers parallel to (110) (Fig. 2). As each cobalt atom is connected to four others, the underlying topology of the layer is a (4,4) re­cta­ngular grid. The inter­nuclear Co···Co through-space distances across the grid apertures are 13.17 Å and 25.14 Å.

### 3. Supra­molecular features

Individual [Co(hqda)(4-bpmp)(H_2_O)_2_]_n_ layers stack in a *AAA* pattern along the *a* crystal direction (Fig. 3). The supra­molecular O—H···O hydrogen bonding between aqua ligands in one layer and ligated hqda O atoms in two others provides the impetus for the formation of the three-dimensional crystal structure of the title compound.

Disordered water molecules of crystallization occupy incipient channels along [1 0 0]. These could not be refined well, and thus their electron density was modeled using the *SQUEEZE* subroutine of *PLATON* (Spek, 2009). The resulting analysis indicated the presence of approximately five water molecules per unit cell, in a region comprising 20.6% of the total unit cell volume.

### 4. Database survey

This compound was not previously reported in the CCDC.

### 5. Synthesis and crystallization

Cobalt(II) nitrate hexahydrate and hydro­quinone-*O,O'*-di­acetic acid (H_2_hqda) were obtained commercially. Bis(4-pyridymethyl)­piperazine (4-bpmp) was prepared *via* a published procedure (Niu *et al.*, 2001). A mixture of cobalt(II) nitrate hexahydrate (68 mg, 0.23 mmol), H_2_hqda (84 mg, 0.37 mmol), 4-bpmp (99 mg, 0.37 mmol), 0.25 ml of a 1.0 *M* NaOH solution and 10.0 g water (550 mmol) was placed into a 23 ml Teflon-lined Parr acid digestion bomb, which was then heated under autogenous pressure at 393 K for 24 h. Pink plates of the title compound were obtained in a multi-phase mixture.

### 6. Refinement

All H atoms bound to C atoms were placed in calculated positions, with C—H = 0.95–0.99 Å, and refined in riding mode with *U*_iso_ = 1.2*U*_eq_(C). The H atoms within the aqua ligand were found in a difference Fourier map, restrained with O—H = 0.85 Å and refined with *U*_iso_ = 1.5*U*_eq_(O).

### Crystal data

**Table:**

[Co(C_10_H_8_O_6_)(C_16_H_20_N_4_)(H_2_O)_2_]·5H_2_O	*Z* = 1
*M**_r_* = 677.57	*F*(000) = 357
Triclinic, *P*1	*D*_x_ = 1.475 Mg m^−^^3^
*a* = 5.7727 (8) Å	Mo *K*α radiation, λ = 0.71073 Å
*b* = 10.3421 (15) Å	Cell parameters from 2300 reflections
*c* = 13.1675 (19) Å	θ = 2.5–25.0°
α = 87.175 (2)°	µ = 0.63 mm^−^^1^
β = 78.856 (2)°	*T* = 173 K
γ = 81.474 (2)°	Plate, pink
*V* = 762.61 (19) Å^3^	0.19 × 0.17 × 0.05 mm

### Data collection

**Table:**

Bruker APEXII CCD diffractometer	2786 independent reflections
Radiation source: fine-focus sealed tube	2032 reflections with *I* > 2σ(*I*)
Graphite monochromator	*R*_int_ = 0.053
φ and ω scans	θ_max_ = 25.4°, θ_min_ = 2.0°
Absorption correction: multi-scan (TWINABS; Sheldrick, 2003)	*h* = −6→6
*T*_min_ = 0.676, *T*_max_ = 0.745	*k* = −12→12
13385 measured reflections	*l* = 0→15

### Refinement

**Table:**

Refinement on *F*^2^	Primary atom site location: structure-invariant direct methods
Least-squares matrix: full	Secondary atom site location: difference Fourier map
*R*[*F*^2^ > 2σ(*F*^2^)] = 0.093	Hydrogen site location: inferred from neighbouring sites
*wR*(*F*^2^) = 0.228	H-atom parameters constrained
*S* = 1.10	*w* = 1/[σ^2^(*F*_o_^2^) + (0.0164*P*)^2^ + 9.3*P*] where *P* = (*F*_o_^2^ + 2*F*_c_^2^)/3
2786 reflections	(Δ/σ)_max_ < 0.001
179 parameters	Δρ_max_ = 0.71 e Å^−^^3^
0 restraints	Δρ_min_ = −0.89 e Å^−^^3^

### Special details

**Table:**

Experimental. TWINABS-2012/1 (Bruker,2012) was used for absorption correction.For component 1: wR2(int) was 0.0549 before and 0.0460 after correction. The Ratio of minimum to maximum transmission is 0.91. The λ/2 correction factor is Not presentFor component 2: wR2(int) was 0.0664 before and 0.0469 after correction. The Ratio of minimum to maximum transmission not present. The λ/2 correction factor is Not presentFinal HKLF 4 output contains 13385 reflections, Rint = 0.0533 (6718 with I > 3sig(I), Rint = 0.0400)
Geometry. All e.s.d.'s (except the e.s.d. in the dihedral angle between two l.s. planes) are estimated using the full covariance matrix. The cell e.s.d.'s are taken into account individually in the estimation of e.s.d.'s in distances, angles and torsion angles; correlations between e.s.d.'s in cell parameters are only used when they are defined by crystal symmetry. An approximate (isotropic) treatment of cell e.s.d.'s is used for estimating e.s.d.'s involving l.s. planes.
Refinement. Refinement of *F*^2^ against ALL reflections. The weighted *R*-factor *wR* and goodness of fit *S* are based on *F*^2^, conventional *R*-factors *R* are based on *F*, with *F* set to zero for negative *F*^2^. The threshold expression of *F*^2^ > σ(*F*^2^) is used only for calculating *R*-factors(gt) *etc*. and is not relevant to the choice of reflections for refinement. *R*-factors based on *F*^2^ are statistically about twice as large as those based on *F*, and *R*- factors based on ALL data will be even larger.

### Fractional atomic coordinates and isotropic or equivalent isotropic displacement parameters (Å^2^)

**Table:**

	*x*	*y*	*z*	*U*_iso_*/*U*_eq_	
Co1	0.5000	0.0000	0.0000	0.0217 (4)	
O4	0.8245 (8)	0.0527 (5)	−0.0875 (4)	0.0259 (11)	
H4A	0.9116	0.0861	−0.0468	0.039*	
H4B	0.7974	0.1148	−0.1372	0.039*	
O1	0.2947 (8)	0.1293 (4)	−0.0851 (4)	0.0284 (12)	
O2	0.5742 (10)	0.1838 (6)	−0.2164 (4)	0.0437 (15)	
O3	−0.0339 (10)	0.3206 (5)	−0.1427 (5)	0.0442 (15)	
N1	0.4646 (10)	0.1467 (5)	0.1144 (5)	0.0262 (14)	
N2	0.1474 (11)	0.4777 (6)	0.4001 (5)	0.0335 (15)	
C13	0.3664 (14)	0.1882 (7)	−0.1687 (6)	0.0318 (18)	
C12	0.1723 (15)	0.2740 (8)	−0.2163 (7)	0.043 (2)	
H12A	0.2401	0.3498	−0.2528	0.052*	
H12B	0.1244	0.2227	−0.2684	0.052*	
C10	−0.0057 (15)	0.4091 (8)	−0.0716 (8)	0.042 (2)	
C9	−0.2010 (14)	0.4424 (8)	0.0052 (8)	0.042 (2)	
H9	−0.3397	0.4018	0.0088	0.051*	
C5	0.6335 (14)	0.1602 (8)	0.1688 (7)	0.038 (2)	
H5	0.7780	0.1009	0.1571	0.046*	
C1	0.2635 (13)	0.2314 (7)	0.1348 (6)	0.0335 (18)	
H1	0.1390	0.2231	0.0988	0.040*	
C6	0.3746 (14)	0.4588 (9)	0.3307 (6)	0.0380 (19)	
H6A	0.5028	0.4440	0.3719	0.046*	
H6B	0.3944	0.5396	0.2883	0.046*	
C8	0.1075 (14)	0.6027 (8)	0.4482 (6)	0.0378 (19)	
H8A	0.1156	0.6725	0.3939	0.045*	
H8B	0.2350	0.6077	0.4878	0.045*	
C7	0.1316 (15)	0.3748 (8)	0.4801 (6)	0.040 (2)	
H7A	0.2605	0.3745	0.5200	0.048*	
H7B	0.1524	0.2886	0.4475	0.048*	
C11	0.1974 (15)	0.4660 (8)	−0.0770 (7)	0.042 (2)	
H11	0.3332	0.4424	−0.1295	0.051*	
C2	0.2273 (13)	0.3304 (7)	0.2053 (6)	0.0305 (17)	
H2	0.0812	0.3883	0.2166	0.037*	
C3	0.4032 (13)	0.3448 (7)	0.2590 (6)	0.0301 (17)	
C4	0.6070 (14)	0.2550 (8)	0.2401 (7)	0.043 (2)	
H4	0.7311	0.2593	0.2773	0.051*	

### Atomic displacement parameters (Å^2^)

**Table:**

	*U*^11^	*U*^22^	*U*^33^	*U*^12^	*U*^13^	*U*^23^
Co1	0.0171 (7)	0.0232 (7)	0.0226 (8)	0.0000 (5)	−0.0008 (5)	−0.0004 (5)
O4	0.022 (3)	0.031 (3)	0.025 (3)	−0.006 (2)	−0.003 (2)	0.008 (2)
O1	0.027 (3)	0.022 (2)	0.036 (3)	0.002 (2)	−0.011 (2)	0.003 (2)
O2	0.036 (3)	0.059 (4)	0.035 (3)	−0.018 (3)	−0.001 (3)	0.022 (3)
O3	0.035 (3)	0.037 (3)	0.066 (4)	−0.006 (3)	−0.024 (3)	0.007 (3)
N1	0.021 (3)	0.023 (3)	0.031 (4)	0.001 (2)	0.000 (3)	−0.002 (3)
N2	0.036 (4)	0.035 (4)	0.026 (4)	0.001 (3)	−0.001 (3)	−0.004 (3)
C13	0.037 (5)	0.033 (4)	0.027 (4)	−0.010 (4)	−0.009 (4)	0.007 (3)
C12	0.046 (5)	0.043 (5)	0.046 (6)	−0.021 (4)	−0.017 (4)	0.028 (4)
C10	0.036 (5)	0.027 (4)	0.070 (7)	−0.010 (4)	−0.024 (4)	0.019 (4)
C9	0.023 (4)	0.028 (4)	0.078 (7)	−0.004 (3)	−0.019 (4)	0.012 (4)
C5	0.028 (4)	0.041 (5)	0.047 (5)	0.004 (4)	−0.012 (4)	−0.013 (4)
C1	0.027 (4)	0.034 (4)	0.037 (5)	0.009 (3)	−0.010 (3)	−0.003 (3)
C6	0.035 (4)	0.054 (5)	0.025 (4)	−0.010 (4)	0.000 (3)	−0.012 (4)
C8	0.035 (5)	0.040 (5)	0.034 (5)	0.001 (4)	−0.001 (4)	0.004 (4)
C7	0.038 (5)	0.040 (5)	0.039 (5)	0.014 (4)	−0.012 (4)	−0.009 (4)
C11	0.033 (5)	0.035 (5)	0.059 (6)	−0.004 (4)	−0.010 (4)	0.009 (4)
C2	0.024 (4)	0.033 (4)	0.030 (4)	0.008 (3)	−0.001 (3)	−0.002 (3)
C3	0.027 (4)	0.034 (4)	0.025 (4)	−0.008 (3)	0.008 (3)	0.000 (3)
C4	0.027 (4)	0.045 (5)	0.058 (6)	0.002 (4)	−0.012 (4)	−0.019 (4)

### Geometric parameters (Å, º)

**Table:**

Co1—O4	2.131 (4)	C9—H9	0.9500
Co1—O4^i^	2.131 (4)	C9—C11^ii^	1.375 (12)
Co1—O1	2.084 (5)	C5—H5	0.9500
Co1—O1^i^	2.084 (5)	C5—C4	1.363 (11)
Co1—N1	2.151 (6)	C1—H1	0.9500
Co1—N1^i^	2.151 (6)	C1—C2	1.382 (10)
O4—H4A	0.9131	C6—H6A	0.9900
O4—H4B	0.9130	C6—H6B	0.9900
O1—C13	1.260 (9)	C6—C3	1.517 (10)
O2—C13	1.237 (9)	C8—H8A	0.9900
O3—C12	1.421 (11)	C8—H8B	0.9900
O3—C10	1.388 (10)	C8—C7^iii^	1.508 (11)
N1—C5	1.343 (10)	C7—C8^iii^	1.508 (11)
N1—C1	1.337 (9)	C7—H7A	0.9900
N2—C6	1.440 (10)	C7—H7B	0.9900
N2—C8	1.435 (10)	C11—C9^ii^	1.375 (12)
N2—C7	1.461 (10)	C11—H11	0.9500
C13—C12	1.535 (11)	C2—H2	0.9500
C12—H12A	0.9900	C2—C3	1.373 (11)
C12—H12B	0.9900	C3—C4	1.376 (11)
C10—C9	1.376 (13)	C4—H4	0.9500
C10—C11	1.377 (11)		
			
O4—Co1—O4^i^	180.0	C11^ii^—C9—C10	120.6 (8)
O4—Co1—N1^i^	85.7 (2)	C11^ii^—C9—H9	119.7
O4^i^—Co1—N1^i^	94.3 (2)	N1—C5—H5	118.5
O4—Co1—N1	94.3 (2)	N1—C5—C4	123.0 (7)
O4^i^—Co1—N1	85.7 (2)	C4—C5—H5	118.5
O1^i^—Co1—O4	87.77 (19)	N1—C1—H1	118.2
O1—Co1—O4^i^	87.77 (18)	N1—C1—C2	123.5 (7)
O1^i^—Co1—O4^i^	92.23 (19)	C2—C1—H1	118.2
O1—Co1—O4	92.23 (19)	N2—C6—H6A	108.9
O1^i^—Co1—O1	180.0	N2—C6—H6B	108.9
O1^i^—Co1—N1^i^	90.1 (2)	N2—C6—C3	113.4 (7)
O1—Co1—N1	90.1 (2)	H6A—C6—H6B	107.7
O1^i^—Co1—N1	89.9 (2)	C3—C6—H6A	108.9
O1—Co1—N1^i^	89.9 (2)	C3—C6—H6B	108.9
N1—Co1—N1^i^	180.0 (3)	N2—C8—H8A	109.3
Co1—O4—H4A	112.0	N2—C8—H8B	109.3
Co1—O4—H4B	111.7	N2—C8—C7^iii^	111.8 (7)
H4A—O4—H4B	106.8	H8A—C8—H8B	107.9
C13—O1—Co1	127.2 (5)	C7^iii^—C8—H8A	109.3
C10—O3—C12	116.7 (7)	C7^iii^—C8—H8B	109.3
C5—N1—Co1	124.3 (5)	N2—C7—C8^iii^	110.0 (6)
C1—N1—Co1	119.6 (5)	N2—C7—H7A	109.7
C1—N1—C5	116.2 (6)	N2—C7—H7B	109.7
C6—N2—C7	111.1 (6)	C8^iii^—C7—H7A	109.7
C8—N2—C6	110.9 (7)	C8^iii^—C7—H7B	109.7
C8—N2—C7	109.3 (6)	H7A—C7—H7B	108.2
O1—C13—C12	115.8 (7)	C10—C11—H11	120.5
O2—C13—O1	127.3 (7)	C9^ii^—C11—C10	119.1 (9)
O2—C13—C12	116.9 (7)	C9^ii^—C11—H11	120.5
O3—C12—C13	113.7 (7)	C1—C2—H2	120.1
O3—C12—H12A	108.8	C3—C2—C1	119.8 (7)
O3—C12—H12B	108.8	C3—C2—H2	120.1
C13—C12—H12A	108.8	C2—C3—C6	120.6 (7)
C13—C12—H12B	108.8	C2—C3—C4	116.6 (7)
H12A—C12—H12B	107.7	C4—C3—C6	122.8 (7)
C9—C10—O3	115.6 (7)	C5—C4—C3	121.0 (8)
C9—C10—C11	120.3 (9)	C5—C4—H4	119.5
C11—C10—O3	124.1 (9)	C3—C4—H4	119.5
C10—C9—H9	119.7		
			
Co1—O1—C13—O2	−1.8 (12)	N1—C1—C2—C3	−0.3 (12)
Co1—O1—C13—C12	177.7 (5)	N2—C6—C3—C2	48.1 (10)
Co1—N1—C5—C4	179.3 (7)	N2—C6—C3—C4	−135.2 (8)
Co1—N1—C1—C2	−178.6 (6)	C12—O3—C10—C9	−173.6 (7)
O4—Co1—O1—C13	12.3 (6)	C12—O3—C10—C11	8.8 (11)
O4^i^—Co1—O1—C13	−167.7 (6)	C10—O3—C12—C13	66.9 (9)
O4^i^—Co1—N1—C5	125.5 (6)	C9—C10—C11—C9^ii^	−0.8 (13)
O4—Co1—N1—C5	−54.5 (6)	C5—N1—C1—C2	1.5 (12)
O4—Co1—N1—C1	125.6 (6)	C1—N1—C5—C4	−0.8 (12)
O4^i^—Co1—N1—C1	−54.4 (6)	C1—C2—C3—C6	175.3 (7)
O1^i^—Co1—N1—C5	33.2 (6)	C1—C2—C3—C4	−1.6 (12)
O1—Co1—N1—C5	−146.8 (6)	C6—N2—C8—C7^iii^	179.1 (7)
O1—Co1—N1—C1	33.3 (6)	C6—N2—C7—C8^iii^	179.8 (7)
O1^i^—Co1—N1—C1	−146.7 (6)	C6—C3—C4—C5	−174.6 (8)
O1—C13—C12—O3	26.2 (10)	C8—N2—C6—C3	−166.4 (7)
O2—C13—C12—O3	−154.2 (7)	C8—N2—C7—C8^iii^	57.0 (10)
O3—C10—C9—C11^ii^	−176.9 (7)	C7—N2—C6—C3	71.8 (9)
O3—C10—C11—C9^ii^	176.7 (7)	C7—N2—C8—C7^iii^	−58.1 (9)
N1—Co1—O1—C13	106.6 (6)	C11—C10—C9—C11^ii^	0.8 (13)
N1^i^—Co1—O1—C13	−73.4 (6)	C2—C3—C4—C5	2.2 (13)
N1—C5—C4—C3	−1.1 (14)		

Symmetry codes: (i) −*x*+1, −*y*, −*z*; (ii) −*x*, −*y*+1, −*z*; (iii) −*x*, −*y*+1, −*z*+1.

### Hydrogen-bond geometry (Å, º)

**Table:**

*D*—H···*A*	*D*—H	H···*A*	*D*···*A*	*D*—H···*A*
O4—H4*A*···O1^iv^	0.91	2.28	2.945 (7)	130
O4—H4*B*···O2	0.91	1.85	2.636 (7)	143

Symmetry code: (iv) *x*+1, *y*, *z*.
